# Supplementary material for: Unsupervised Hierarchical Clustering of Head and Neck Cancer Patients by Pre-Treatment Plasma Metabolomics Creates Prognostic Metabolic Subtypes
Source: Cancers (Basel). 2023 Jun 14;15(12):3184. doi: 10.3390/cancers15123184 (PMC10296258; doi:10.3390/cancers15123184)
Supplement: Supplementary file 1 [file cancers-15-03184-s001.zip › cancers-2283304-supplementary.pdf]

Supplementary Tables and Figures.

Supplementary Table S1. List of 186 lab-confirmed metabolites used in the hierarchical clustering.
Supplementary Table 1 is provided as an excel file

Supplementary Table S2. List of metabolic pathways tested for enrichment. The table shows the number of metabolites affiliated with each pathway that were among the 38 associated with the cluster subtypes at FDR<0.00001 relative to the 186 total metabolites used for clustering

SMPDB metabolic pathways	# that met p-adjust<0.00001	# in total	P value
Fatty Acid Biosynthesis	4	6	0.004
Transfer of Acetyl Groups into Mitochondria	3	5	0.03
Arginine and Proline Metabolism	5	12	0.06
Galactose Metabolism	3	6	0.07
Ammonia Recycling	0	9	0.12
Glutamate Metabolism	0	9	0.12
Betaine Metabolism	2	4	0.14
Lactose Degradation	2	4	0.14
Phosphatidylcholine Biosynthesis	2	4	0.14
Beta-Alanine Metabolism	0	7	0.17
Purine Metabolism	0	7	0.17
Alanine Metabolism	0	6	0.21
Alpha Linolenic Acid and Linoleic Acid Metabolism	2	5	0.27
Spermidine and Spermine Biosynthesis	2	5	0.27
Cysteine Metabolism	0	4	0.31
Glutathione Metabolism	0	4	0.31
Steroidogenesis	0	4	0.31
Methionine Metabolism	3	9	0.33
Tryptophan Metabolism	1	11	0.34
Arachidonic Acid Metabolism	0	3	0.38
Mitochondrial Beta-Oxidation of Short Chain Saturated Fatty Acids	0	3	0.38
Phytanic Acid Peroxisomal Oxidation	0	3	0.38
Propanoate Metabolism	0	3	0.38
Pyrimidine Metabolism	0	3	0.38
Selenoamino Acid Metabolism	0	3	0.38
Vitamin B6 Metabolism	0	3	0.38
Citric Acid Cycle	2	6	0.43
Oxidation of Branched Chain Fatty Acids	2	6	0.43
Warburg Effect	3	10	0.44
Valine, Leucine, and Isoleucine Degradation	1	9	0.48
Catecholamine Biosynthesis	1	3	0.58
Fructose and Mannose Degradation	1	3	0.58
Lactose Synthesis	1	3	0.58

Methylhistidine Metabolism	1	3	0.58
Starch and Sucrose Metabolism	1	3	0.58
Amino Sugar Metabolism	2	7	0.59
Histidine Metabolism	2	7	0.59
Sphingolipid Metabolism	2	7	0.59
Bile Acid Biosynthesis	1	7	0.68
Nicotinate and Nicotinamide Metabolism	1	7	0.68
Glycine and Serine Metabolism	4	17	0.74
Carnitine Synthesis	2	8	0.74
Urea Cycle	2	8	0.74
Glycerolipid Metabolism	1	4	0.82
Glycolysis	1	4	0.82
Lysine Degradation	1	4	0.82
Phosphatidylethanolamine Biosynthesis	1	4	0.82
Pyruvate Metabolism	1	4	0.82
Steroid Biosynthesis	1	4	0.82
Tyrosine Metabolism	2	9	0.89
Aspartate Metabolism	1	5	0.98
Beta Oxidation of Very Long Chain Fatty Acids	1	5	0.98
Gluconeogenesis	1	5	0.98
Glucose-Alanine Cycle	1	5	0.98
Phenylalanine and Tyrosine Metabolism	1	5	0.98
Butyrate Metabolism	1	2	*
Caffeine Metabolism	0	2	*
Fatty acid Metabolism	1	2	*
Folate Metabolism	0	2	*
Homocysteine Degradation	1	2	*
Ketone Body Metabolism	1	2	*
Malate-Aspartate Shuttle	0	2	*
Mitochondrial Beta-Oxidation of Long Chain Saturated Fatty Acids	0	2	*
Mitochondrial Electron Transport Chain	0	2	*
Nucleotide Sugars Metabolism	1	2	*
Pantothenate and CoA Biosynthesis	0	2	*
Pentose Phosphate Pathway	1	2	*
Phenylacetate Metabolism	0	2	*
Phospholipid Biosynthesis	1	2	*
Pyruvaldehyde Degradation	0	2	*
Retinol Metabolism	0	2	*
Taurine and Hypotaurine Metabolism	0	2	*
Threonine and 2-Oxobutanoate Degradation	0	2	*
Biotin Metabolism	0	1	*
Estrone Metabolism	1	1	*
Fatty Acid Elongation in Mitochondria	1	1	*

Inositol Metabolism	0	1	*
Inositol Phosphate Metabolism	0	1	*
Mitochondrial Beta-Oxidation of Medium Chain Saturated Fatty Acids	0	1	*
Phosphatidylinositol Phosphate Metabolism	0	1	*
Plasmalogen Synthesis	0	1	*
Porphyrin Metabolism	0	1	*
Riboflavin Metabolism	0	1	*
Sulfate/Sulfite Metabolism	0	1	*
Thiamine Metabolism	0	1	*
Thyroid hormone synthesis	0	1	*
Trehalose Degradation	0	1	*
Ubiquinone Biosynthesis	1	1	*

*Only pathways with ≥3 metabolites were considered for enrichment analysis
Chi-squared P values were calculated by comparing the number of pathway hits from the 38 metabolites at FDR<0.00001 relative to how many would be expected by chance from the 186 total via a 2x2 table

Supplementary Table S3. A Pearson's correlation table of 186 metabolites corresponding to the clustered heatmap of Supplementary Figure S3.

Supplementary Table S3 is provided as an excel file.

Supplementary Table S4. Clustering metrics used to determine the optimal number of patient clusters in the hierarchical clustering model of 209 head and neck cancer patients using 189 plasma metabolites

	Number of Clusters				
	2	3	4	5	6
Bayesian Information Criterion[a]	38,523	38,523	38,464	38,563	38,748
Average silhoutte score[b]	0.05	0.03	0.03	0.03	0.03
Pearson's γ[a]	0.18	0.26	0.30	0.34	0.35
Dunn index[b]	0.34	0.34	0.34	0.34	0.34
Entropy[a]	0.68	1.02	1.29	1.46	1.50
Calinski-Harabasz index[b]	12.20	9.12	8.32	7.73	7.31

[a]Smaller indicates better clustering
[b]Higher indicates better clustering
Metrics are calculated in JASP and based on the Euclidean distance and Ward.D linkage;
Green highlighting indicates the optimal number of clusters according to that specific metric

Supplementary Table S5. Sensitivity analyses showing the estimated associations of overall survival via Cox models among the ever smoking HNSCC population (n=113, n_{deaths}=37) using two alternative clustering approaches, Random Forest and K-means, each creating two alternative high-risk vs low-risk (referent) metabolic subtypes

	HR	95% CI	P value
**Random Forest clustering**			
Unadjusted model	1.73	(0.89, 3.36)	0.10
Age, sex, HPV, and smoking adj. model	1.67	(0.86, 3.28)	0.13
Fully adjusted model[a]	6.75	(2.24, 20.3)	<0.001
**K-means clustering**			
Unadjusted model	1.73	(0.90, 3.31)	0.10
Age, sex, HPV adj. model	1.90	(0.97, 3.72)	0.06
Fully adjusted model[a]	2.75	(1.09, 6.95)	0.03

[a]Further adjusted for race, body mass index, alcohol history, marital status, tumor site and stage, treatment, Eastern Cooperative Oncology Group (ECOG) performance, gastronomy tube, prior comorbidities, albumin, hemoglobin, neutrophil-to-lymphocyte ratio, and platelet-to-lymphocyte ratio; 25 subjects including 3 deaths were removed due to missing covariates.
HNSCC= head and neck cancer squamous cell carcinoma, HR=hazard ratio, CI=confidence interval, HPV=human papillomavirus

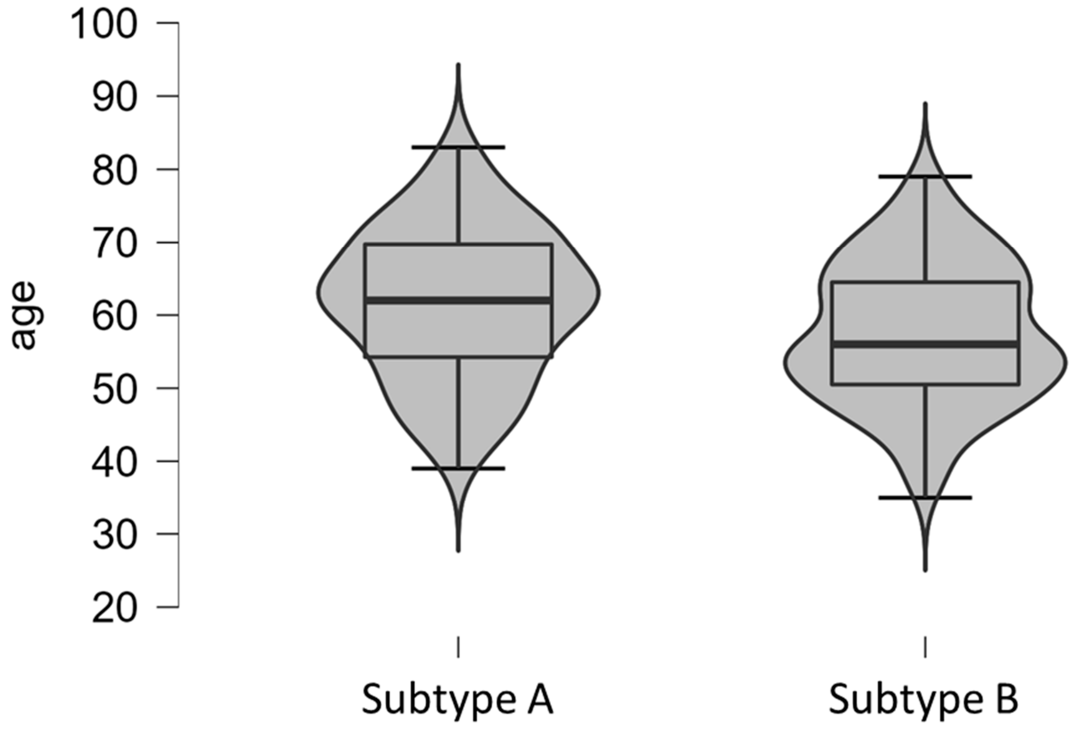

Supplementary Figure S1. A stratified boxplot of the age distribution by metabolic subtype. The average age (standard deviation) of subtypes A and B are 61.7 (10.2) and 57.6 (9.7), respectively; t-test P-value = 0.004. The median age (inter-quartile range) of subtypes A and B are 62 (15.5) and 56 (14); Wilcoxon rank sum P-value = 0.006.

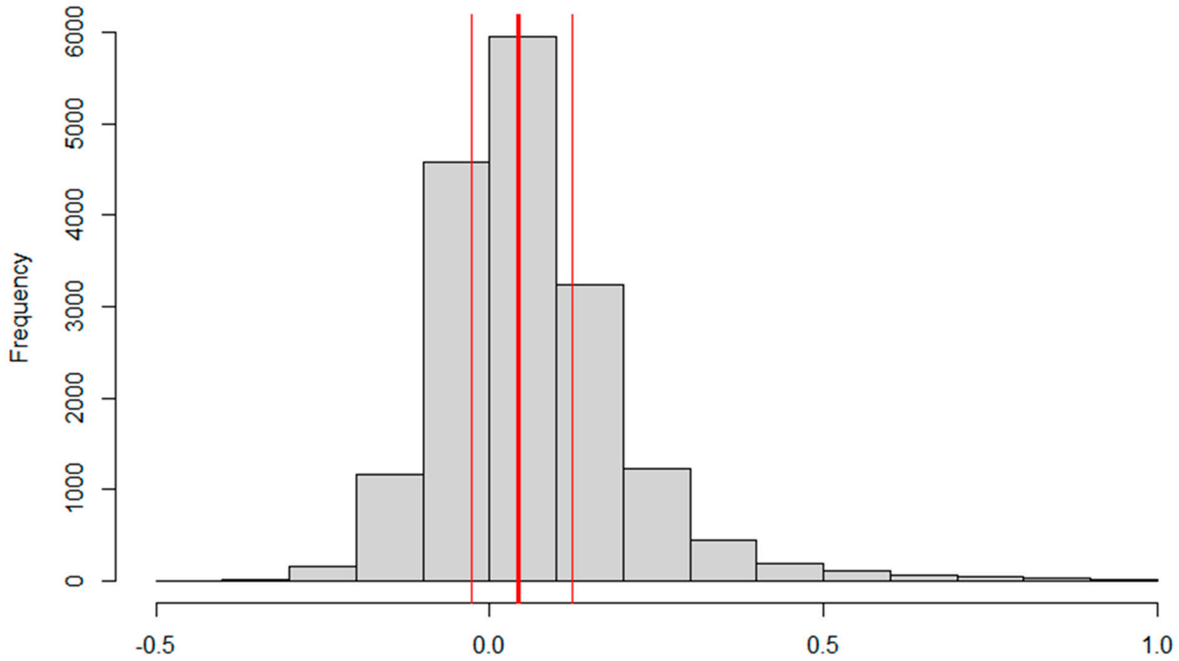

Supplementary Figure S2. A histogram of pair-wise Pearson correlation coefficients between the 186 plasma metabolites used for clustering. The median correlation coefficient (thick red line) was 0.04 with an inter-quartile range (thin red lines) from -0.03 to 0.12 demonstrating generally weak correlation between any two metabolites, suggesting metabolic heterogeneity throughout the total.

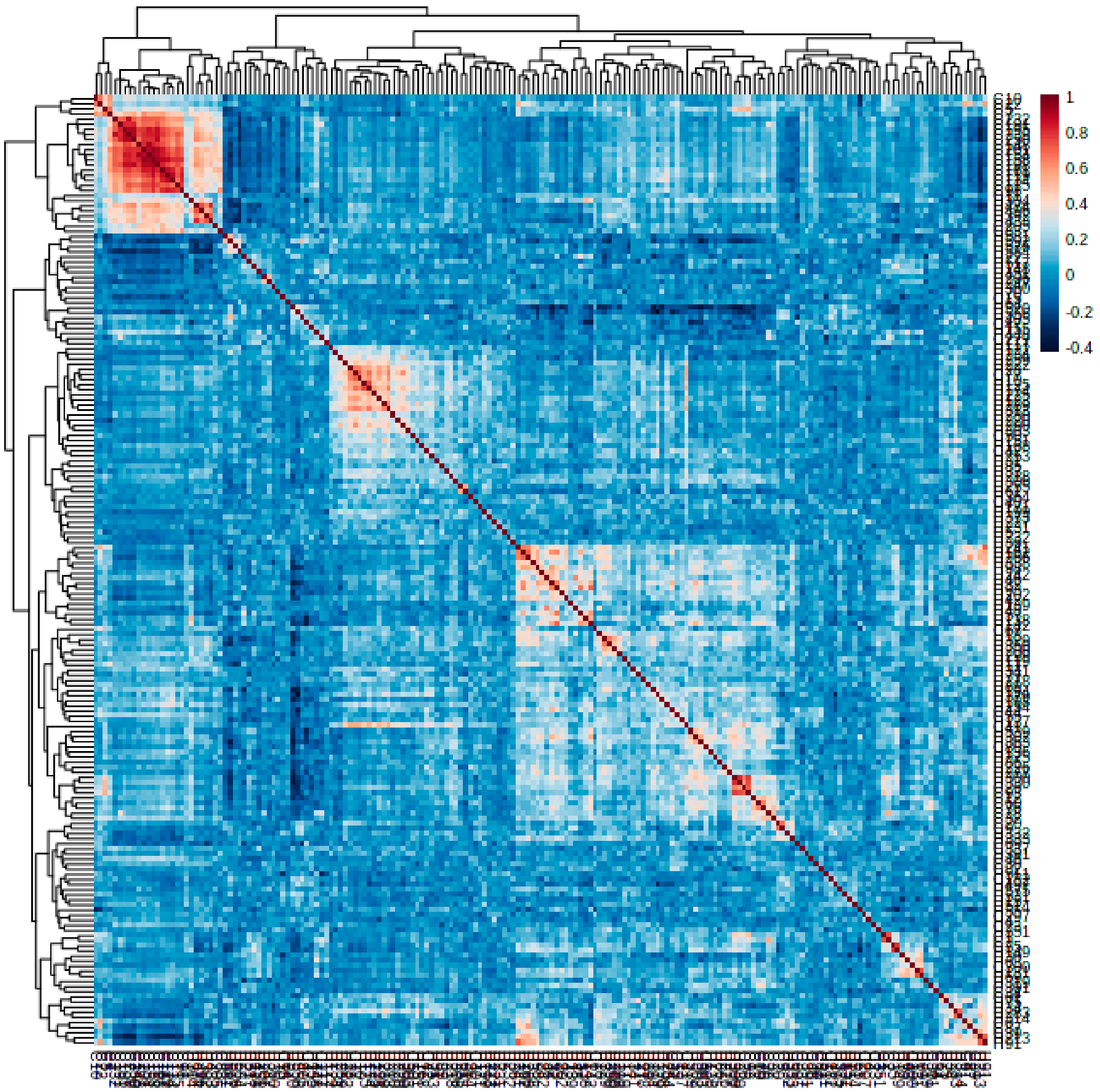

Supplementary Figure S3. A clustered heatmap of the pair-wise Pearson correlation coefficients between the 186 plasma metabolites used for clustering. The red areas indicate that despite the average correlation being low (0.04), there are groups of metabolites that are highly correlated likely because they belong to similar metabolic pathways.

Supplementary Figure S4A-D. Progression-free Kaplan-Meier survival curves by Metabolic Subtype. Panel A is among the full population (n=62 events) in which the estimated 3-year progression-free survival is 68% for subtype A (dark red) and 79% for subtype B (light red); Log-rank p-value = 0.05. Panel B shows the metabolic subtype progression-free survival curves (A vs B) amongst never smokers (n=15 events). The estimated 3-year survival is 93.1% for subtype A, and 82.6% for subtype B; P=0.99. Panel C shows the metabolic subtype progression-free survival curves (A vs B) amongst ever smokers (n=47 events). The estimated 3-year survival is 52.7% for subtype A, and 77.2% for subtype B; P=0.01. Panel D further stratifies the survival curves by HPV status (unrelated and related) amongst ever smokers. The estimated 3-year progresison-free survival for the four groups is 40.6% for Subtype A, HPV-unrelated (dark red); 73.8% for Subtype B, HPV-unrelated (light red); 71.8% for subtype A, HPV-related (dark blue); and 83.3% for subtype B, HPV-related (light blue).

Panel A

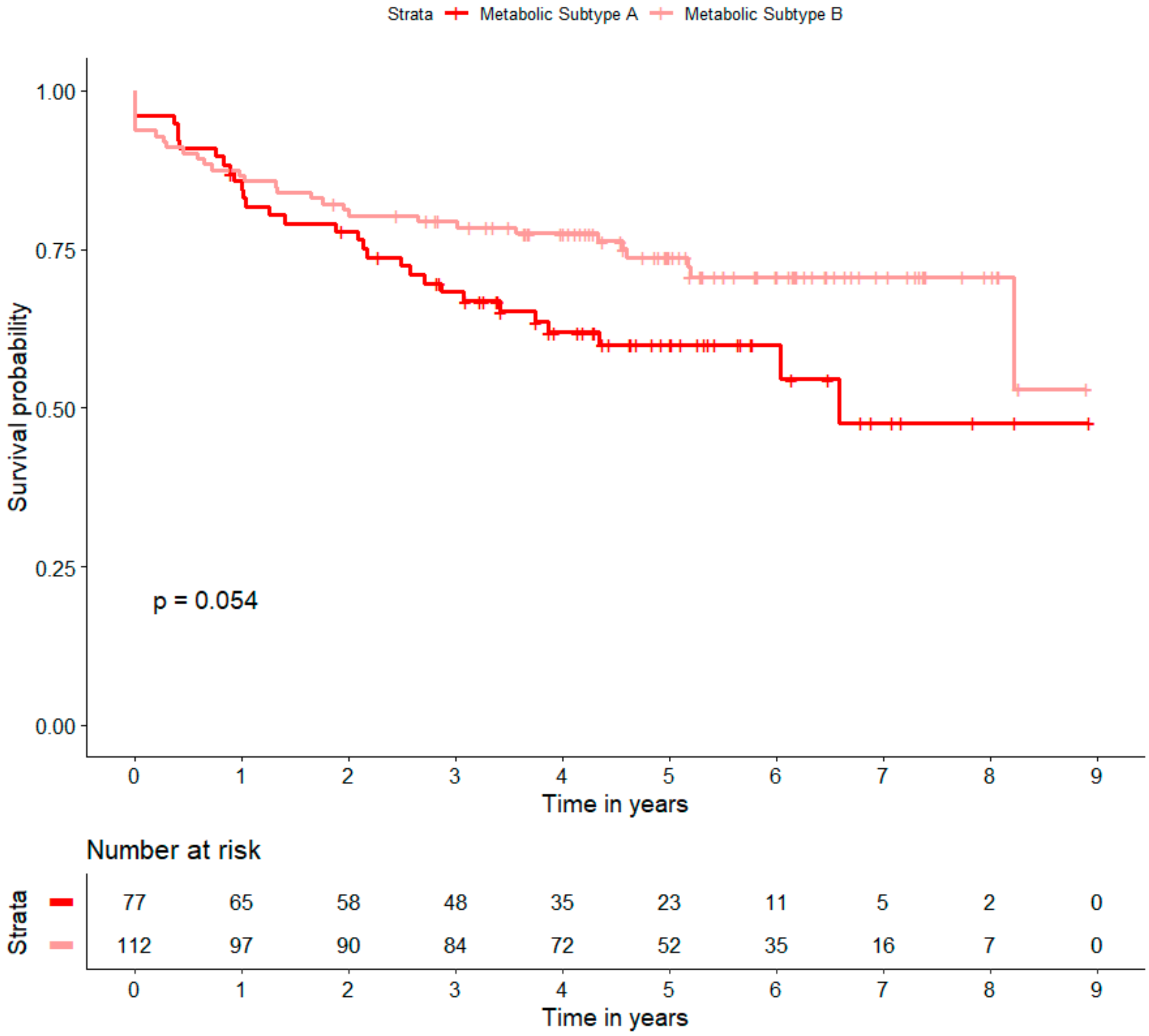

Panel B

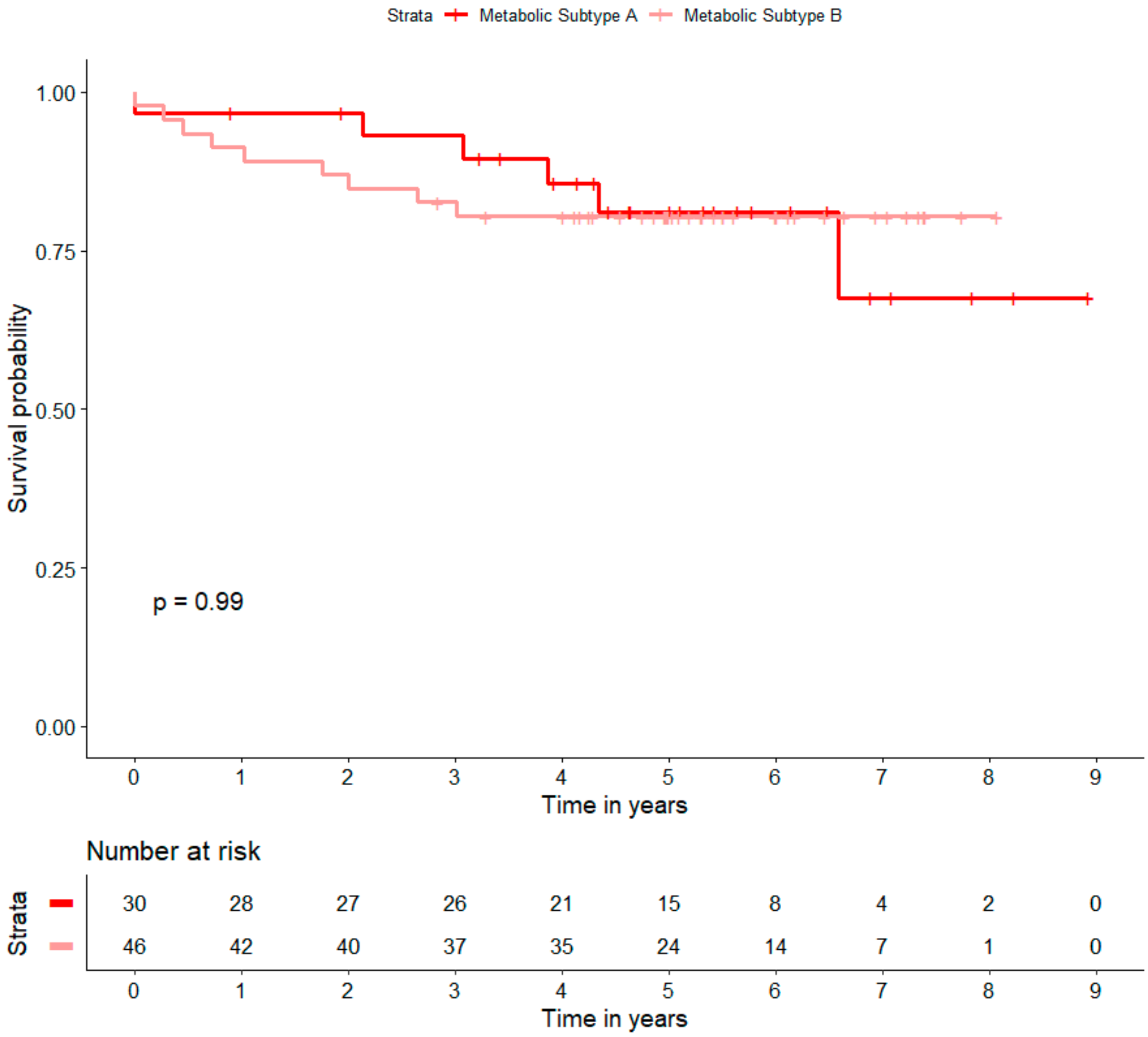
Strata
Metabolic Subtype A
Metabolic Subtype B
Survival probability
1.00
0.75
0.50
0.25
0.00
p = 0.99
0
1
2
3
4
5
6
7
8
9
Time in years
Number at risk
Strata
30
28
27
26
21
15
8
4
2
0
46
42
40
37
35
24
14
7
1
0
0
1
2
3
4
5
6
7
8
9
Time in years

Panel C

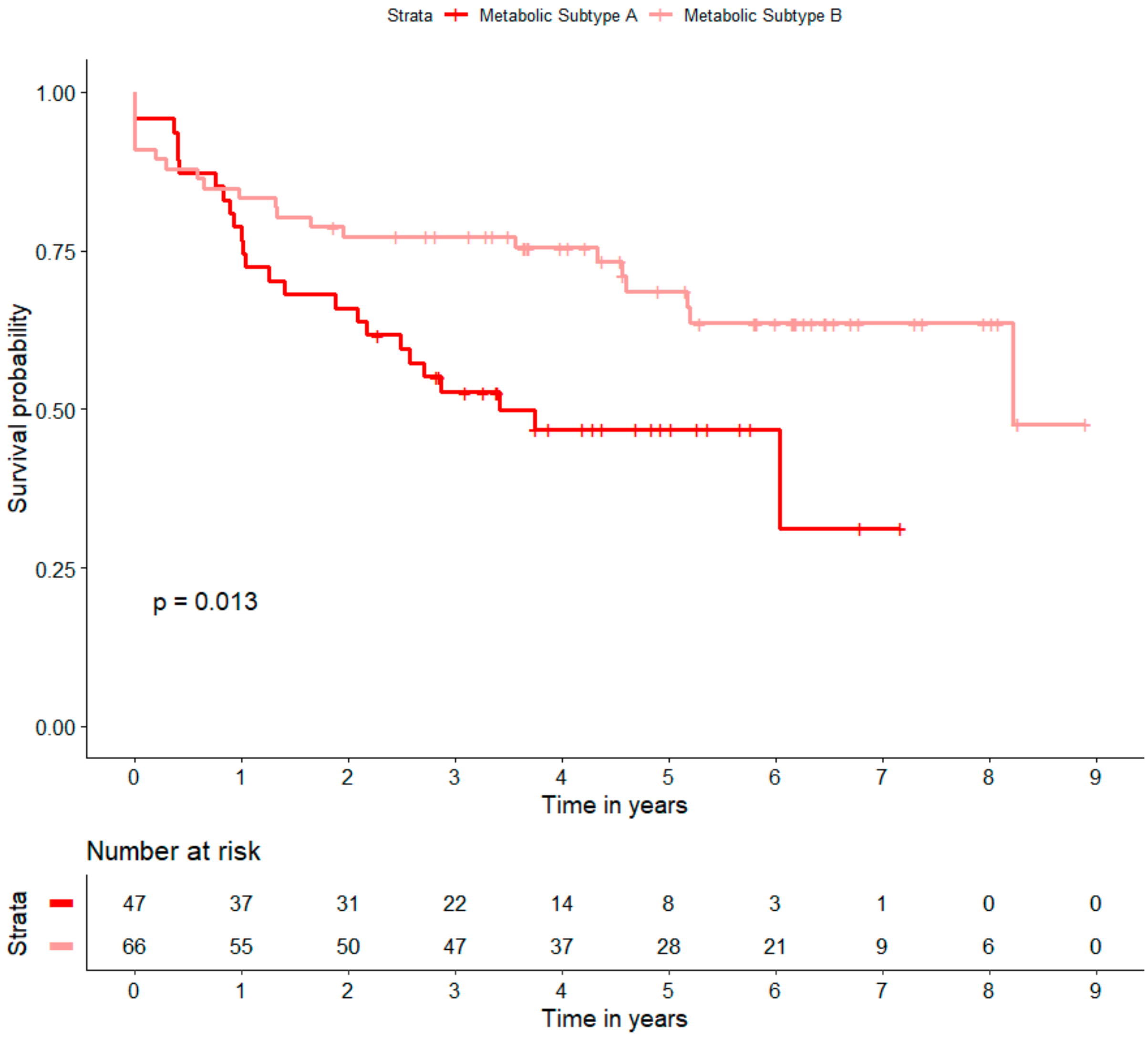

Strata
Metabolic Subtype A
Metabolic Subtype B
Survival probability
1.00
0.75
0.50
0.25
0.00
p = 0.013
0
1
2
3
4
5
6
7
8
9
Time in years
Number at risk
Strata
47 37 31 22 14 8 3 1 0 0
66 55 50 47 37 28 21 9 6 0
0
1
2
3
4
5
6
7
8
9
Time in years

Panel D

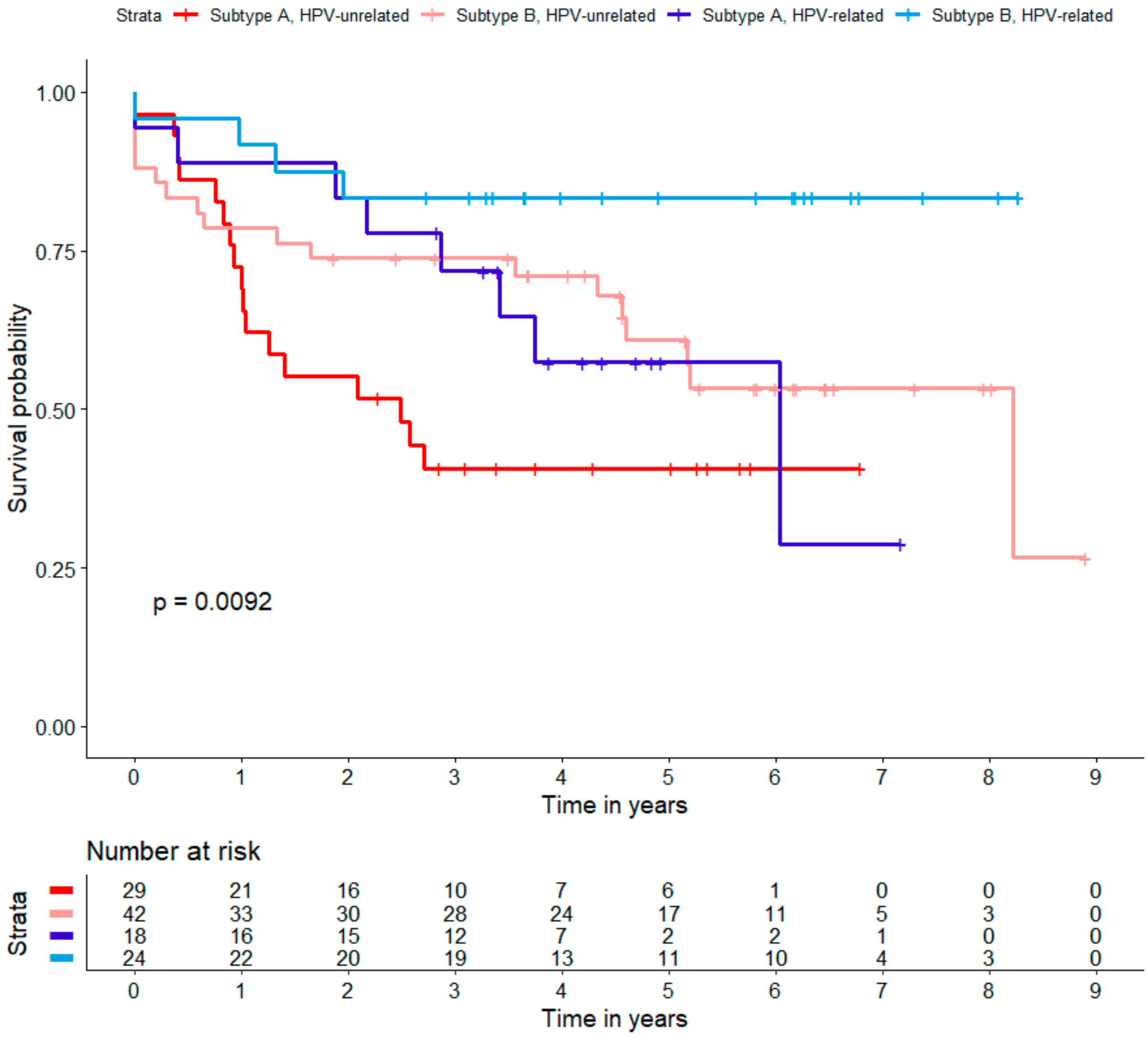

Strata
Subtype A, HPV-unrelated
Subtype B, HPV-unrelated
Subtype A, HPV-related
Subtype B, HPV-related
Survival probability
1.00
0.75
0.50
0.25
0.00
p = 0.0092
0
1
2
3
4
5
6
7
8
9
Time in years
Number at risk
Strata
29 21 16 10 7 6 1 0 0 0
42 33 30 28 24 17 11 5 3 0
18 16 15 12 7 2 2 1 0 0
24 22 20 19 13 11 10 4 3 0
0
1
2
3
4
5
6
7
8
9
Time in years
